# Features of ZED1227: The First-In-Class Tissue Transglutaminase Inhibitor Undergoing Clinical Evaluation for the Treatment of Celiac Disease

**DOI:** 10.3390/cells11101667

**Published:** 2022-05-17

**Authors:** Christian Büchold, Martin Hils, Uwe Gerlach, Johannes Weber, Christiane Pelzer, Andreas Heil, Daniel Aeschlimann, Ralf Pasternack

**Affiliations:** 1 Zedira GmbH, Roesslerstrasse 83, 64293 Darmstadt, Germany; buechold@zedira.com (C.B.); hils@zedira.com (M.H.); weber@zedira.com (J.W.); pelzer@zedira.com (C.P.); andyheil@web.de (A.H.); 2 Sanofi-Aventis Deutschland GmbH, UG Serves as External Consultant for Medicinal Chemistry to Zedira, 65926 Frankfurt, Germany; duamger@yahoo.de; 3 Matrix Biology & Tissue Repair Research Unit, School of Dentistry, Cardiff University, Heath Park, Cardiff CF14 4XY, Wales, UK; aeschlimanndp@cardiff.ac.uk

**Keywords:** tissue transglutaminase, transglutaminase inhibitor, celiac disease, drug discovery

## Abstract

ZED1227 is a small molecule tissue transglutaminase (TG2) inhibitor. The compound selectively binds to the active state of TG2, forming a stable covalent bond with the cysteine in its catalytic center. The molecule was designed for the treatment of celiac disease. Celiac disease is an autoimmune-mediated chronic inflammatory condition of the small intestine affecting about 1–2% of people in Caucasian populations. The autoimmune disease is triggered by dietary gluten. Consumption of staple foods containing wheat, barley, or rye leads to destruction of the small intestinal mucosa in genetically susceptible individuals, and this is accompanied by the generation of characteristic TG2 autoantibodies. TG2 plays a causative role in the pathogenesis of celiac disease. Upon activation by Ca^2+^, it catalyzes the deamidation of gliadin peptides as well as the crosslinking of gliadin peptides to TG2 itself. These modified biological structures trigger breaking of oral tolerance to gluten, self-tolerance to TG2, and the activation of cytotoxic immune cells in the gut mucosa. Recently, in an exploratory proof-of-concept study, ZED1227 administration clinically validated TG2 as a “druggable” target in celiac disease. Here, we describe the specific features and profiling data of the drug candidate ZED1227. Further, we give an outlook on TG2 inhibition as a therapeutic approach in indications beyond celiac disease.

## 1. Introduction

Transglutaminases, formally designated as “protein-glutamine: amine-glutamyl transferases” (EC 2.3.2.13), were first described in the 1950s by Heinrich Waelsch and co-workers [1]. Since then, eight active human transglutaminases have been discovered and their roles in a multitude of physiological processes described. Moreover, transglutaminases’ involvement in a variety of disorders has either been conclusively demonstrated or is strongly suspected. Tissue transglutaminase (TG2), which has been the most intensively studied eukaryotic transglutaminase, is one of the most enigmatic enzymes. Besides transamidation and deamidation, phosphorylation and disulfide isomerase activities are also described. Moreover, TG2 acts as a G-protein in signaling pathways involving PLCδ1. TG2 can locate intracellularly in the cytosol, the nucleus, and mitochondria. Extracellularly, TG2 is membrane-associated or present in complexes with select proteins within the extracellular matrix (ECM), especially fibronectin or specific collagens. TG2 is ubiquitously expressed in tissues derived from the different embryonic germ layers and is eventually present in almost every tissue or organ of the body. At high GTP/GDP and low Ca^2+^-concentrations intracellularly, TG2 adopts a compact inactive conformation. In contrast, high Ca^2+^ concentrations in the extracellular environment drive the switch of TG2 into its active form that displays transamidation and deamidation activity. We refer the reader to the following reviews for comprehensive discussions of this subject [2,3].

In terms of disease processes, TG2 has been implicated in several fibrotic disorders, some cancer types, various neurodegenerative disorders, acne, psoriasis, and cataract development [3,4]. However, in many cases it remains unclear whether TG2 plays a causative role within the pathogenesis or is a bystander that “just randomly crosslinks” proteins at an advanced disease state. While the bystander effect in itself can contribute to pathogenesis and thereby exacerbate the condition, targeting this will not resolve the underlying problem. Accordingly, there is the need to develop compounds to validate transglutaminase as a druggable target for a specific physiological context. Drug discovery programs must consider the most promising indication based on the evaluation of scientific and commercial aspects. Therefore, we opted for celiac disease as the lead indication. This was largely due to the strong mechanistic evidence available demonstrating a causal function of TG2 in the disease process.

Celiac disease is characterized by chronic inflammation of the small intestinal mucosa, with a substantial impact on quality of life in affected individuals. In genetically susceptible patients, the intestinal lining is progressively destroyed in response to consumption of gluten and related prolamins from cereals such as wheat, barley, and rye. Classical symptoms include nausea, vomiting, diarrhea, abdominal pain, malabsorption, and failure to thrive in children. Currently, no pharmacological treatment for celiac disease is available. Patients must follow a strict, lifelong gluten-free diet. In practice, such a diet is extremely difficult to follow on a daily basis. Most challenging are situations outside the home, and the non-obvious, hidden gluten present in many convenience products. Inadvertent gluten consumption by patients is a major problem, occurs frequently, and results in permanent mucosal damage [5,6]. Consequently, there is a high medical need for development of adjunct drugs that can support a gluten-free diet and prevent immune activation [7].

The link between celiac disease and wheat had already been identified in the 1940s by the Dutch pediatrician Dicke [8], but the pathogenic mechanism remained unclear. In 1985, transglutaminase activity was shown in the human jejunal mucosa, with increased activity in celiac disease patients [9]. The authors of this study proposed a link between transglutaminase and gliadin in celiac disease. Importantly, in 1997 TG2 was identified as the autoantigen in celiac disease [10]. One year later, selective gliadin deamidation by TG2 resulting in recognition of the respective modified peptides by gut-derived DQ2-restricted T-cells was shown, thus providing insight into the fundamental molecular interplay between TG2 activity, gluten, and the genetic background that leads to activation of the immune system in celiac disease [11,12]. The idea of a pharmacological intervention in celiac disease by TG2-inhibition was suggested at the same time [12]. Today, while some pieces of the puzzle are still missing, a more detailed picture of the early steps of celiac disease pathogenesis has evolved. Active TG2, and its deleterious interaction with gliadin, plays a multifaceted role in celiac disease.

Gliadin, the alcohol soluble fraction of the wheat grain storage protein mixture gluten, is characterized by high proline and glutamine content. Proline residues render gliadin resistant to gastrointestinal proteases as most proteases cannot hydrolyze proline’s substituted and conformationally constrained amide bond. Therefore, digestion of gliadin results in atypically long peptides (e.g., the 26-mer aa 59–84 γ-gliadin peptide and the 33-mer aa 57–89 α-gliadin peptide) potentially reaching the lamina propria [13,14]. Active TG2 in the small intestinal mucosa catalyzes the deamidation of gliadin peptides yielding deamidated gliadin peptides (DGPs), which are negatively charged at key positions [11,12,15]. The DGPs bind with increased affinity to DQ2 or DQ8 MHC class II receptors on antigen-presenting cells (APCs) [13,16]. These DGP presenting DQ2/8-APCs bind to and activate gliadin-specific CD4^+^ T-cells, which may then provide help to DGP-specific B-cells to differentiate into anti-DGP-producing plasma cells [17,18]. Therefore, the DQ2 and DQ8 HLA-alleles, which are present in about 30 to 50% of individuals in Western populations, can be considered as a genetic gate for deamidated gliadin peptides.

The second consequence of TG2 action in the lamina propria is crosslinking of gliadin peptides to themselves, creating hapten-carrier-like gliadin-TG2 complexes [19,20]. For the following immune reactions to develop, gliadin deamidation is mandatory. It is still unclear exactly where gliadin deamidation takes place. It seems plausible that already partially deamidated gliadin is crosslinked to TG2, but deamidation may also take place along the entire cascade until presentation by immune cells. TG2-specific B-cells may recognize and internalize these TG2-DGP complexes, present the DGP bound to MHC and become activated by interaction with gluten-specific T-cells, resulting in TG2 autoantibody producing plasma cells [20,21]. However, several alternative mechanisms for uptake of DGP by TG2-specific B-cells have been proposed: Firstly, TG2 may crosslink gliadin directly to IgD on the surface of B-cells, thus allowing the internalization [22]. Secondly, the incorporation of gliadin in autocatalytically formed multimeric TG2 complexes may occur, which serve as very potent B-cell antigens for anti-TG2 (and anti-DGP) specific B-cells [23]. Finally, an alternative pathway to TG2-gliadin crosslinking is the uptake of the rather stable TG2-gliadin thioester reaction intermediates, which become hydrolyzed in the acidic environment of the endosomes, eventually resulting in deamidated gliadin peptides [20]. In summary, TG2 activity leads to two mature B-cell populations, with specificity for TG2 and DGP. TG2 activity enables efficient B-cell/T-cell collaboration in a DGP-dependent manner that ultimately exceeds the necessary threshold for immune activation, leading to clonal expansion of the respective cell populations. This process constitutes a further, although indirect role (antibodies) for TG2 in the early celiac disease pathogenesis. Conversely, the activated CD4^+^ T-cells are believed to direct the whole orchestra of celiac disease small intestinal inflammation, by the release of inflammatory cytokines (e.g., IFN-γ, IL-21) and stimulation of cytotoxic CD8^+^ T-cells [18]. The complex role of TG2 in celiac disease pathogenesis is comprehensively summarized and illustrated in a review by Ludvig Sollid [18].

It must be mentioned that the exact circumstances under which TG2 is activated and catalyzes the respective reactions driving celiac disease pathology remains a matter of debate. It seems to be possible that shedding of epithelial cells into the intestinal lumen and consequential loss of their integrity leads to passive release and activation of the enzyme. B-cells in Peyer’s patches that express TG2-specific IgD may sample such luminal TG2 and form an important conduit to antigen presentation [24]. Retrograde vesicular transport following binding to the transferrin receptor could potentially also facilitate uptake of TG2 from the gut lumen, as well as gliadin peptides, and ultimately transport across the intestinal epithelium [25]. However, it has also been shown that extracellular ATP-mediated activation of the P2X7 receptor can drive efficient active release of TG2 from primary peripheral blood mononuclear cells (PBMCs), macrophages and other immune cells, as well as some tissue resident cells [26]. This is a particularly interesting finding as the respective “danger signal” pathway modulates both innate and adaptive immune responses, and as it provides for a mechanism that supports simultaneous externalization of TG2 and thioredoxin-1 [26]. In the extracellular environment, TG2 undergoes rapid oxidative inactivation via formation of a disulfide bond at two vicinal cysteine residues (Cys370, Cys371), a reaction that is facilitated by Cys230 (found in TG2 but not in other TGs) and locks the enzyme in an extended conformation [27]. Specifically, as Cys230 is located within Ca^2+^-binding site 1, ion occupancy at this site prevents disulfide bond formation and promotes enzyme activation through further cooperative Ca^2+^-binding at sites 2 and 3 [28]. Hence, Ca^2+^-mediated activation and oxidative inactivation of TG2 are finely balanced, and oxidative inactivation therefore substantially blunts extracellular TG2 activity in many biological contexts. Interestingly, this latent pool of extracellular TG2 can be re-activated by thioredoxin-1 [29]. Hence, an insult or inflammatory context leading to activation of the P2X7 receptor creates an environment that enables sustained extracellular TG2 activity.

The emerging understanding of celiac disease pathogenesis and the apparent central role of TG2 led to the speculation that blocking of TG2 activity with specific and potent small molecule inhibitors might, at least in theory, break the vicious cycle in celiac disease. When looking back to work conducted in the early part of the last decade, we must remember, however, that no drug-like molecules were available at the time to evaluate this hypothesis in clinical trials.

Nevertheless, some reports published on the development of transglutaminase inhibitors should be mentioned to provide the necessary context. Early projects centered around coagulation factor XIII (FXIII, plasma transglutaminase). Remarkably, the first transglutaminase inhibitor was developed by nature. The giant Amazon leech *Haementeria ghilianii* produces Tridegin, a 66-amino-acid-polypeptide inhibitor of FXIII, in its saliva to impair clotting in its prey’s blood [30]. In 1981, a first approach to design chloromethyl ketones as FXIII inhibitors was published by a Bayer scientist [31], followed by oxopropyl thioimidazolium derivatives in the early 1990s by Merck Sharp and Dohme researchers [32,33]. However, due to the lack of overall drug-likeness, none of these compounds was further developed.

In the early 2000s, diazo-5-oxonorleucine (DON)-based TG2 inhibitors were published [34] and compounds using this warhead became commercially available from Zedira for R & D purposes, for instance Z006 (*vide infra*). However, the high reactivity of the DON warhead caused such compounds to be curtailed from clinical trials due to reported dose-limiting toxicity [35]. Therefore, the development program of less electrophilic Michael-acceptor class molecules as transglutaminase inhibitors was initiated by our group, eventually leading to the discovery of ZED1227, becoming the first clinical stage TG2 inhibitor. In an initial gluten challenge study, ZED1227 was shown to be well-tolerated and, importantly, to protect celiac patients from gluten-induced mucosal injury [36]. In addition, the success of this clinical study with ZED1227 validated TG2 as a druggable target. In this article some key features and profiling data of ZED1227 are reported. Furthermore, an outlook on the validation of TG2 as a target for pharmacological interventions beyond celiac disease is provided.

## 2. Materials and Methods

### 2.1. Overview on the Synthesis Route of ZED1227

**Figure 1:**
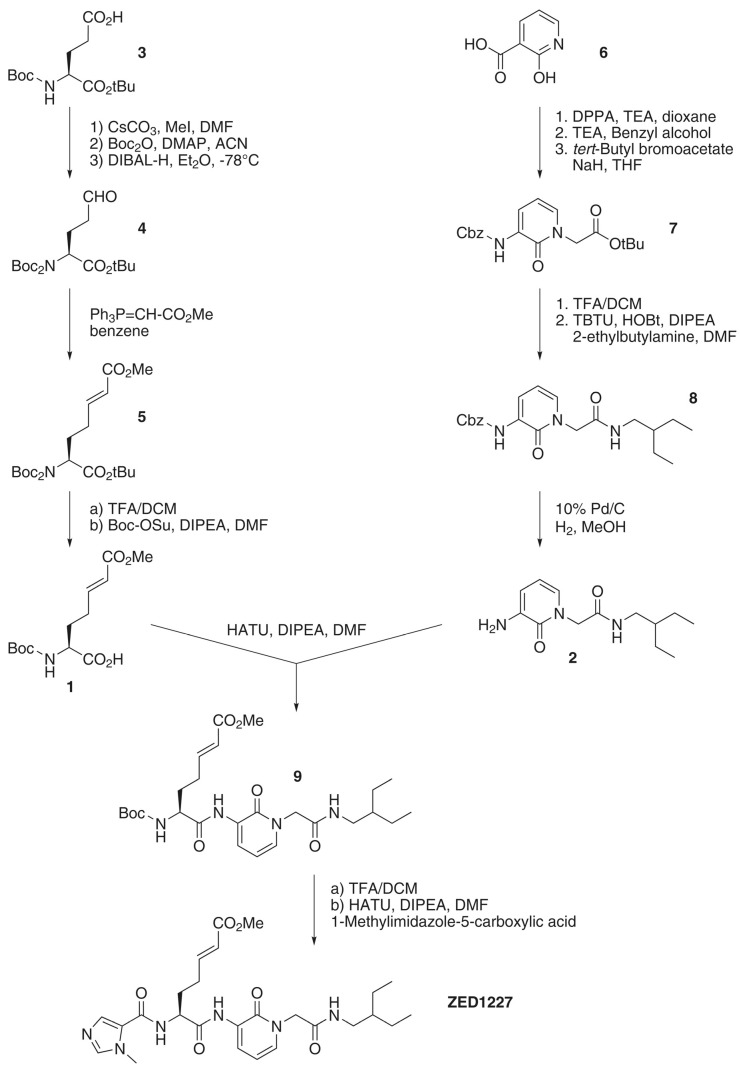
Synthesis scheme of ZED1227. Abbreviations used are included in Appendix A.

The synthesis of ZED1227 consisted of a parallel multi-step synthesis of two building blocks (**1** and **2**). The chiral Michael-acceptor-containing building block (**1**) was obtained by starting with the commercially available Boc (*tert*-butyloxycarbonyl) protected (L)-glutamic acid *tert*-butyl ester (**3**). After methylation of the carboxylic acid side-chain, the introduction of a second *tert*-butyloxycarbonyl protecting group (intermediate compounds not shown in Figure 1) was known to be essential to minimize the nucleophilicity of the nitrogen atom, as otherwise the subsequent reduction leads to a mixture of products [37,38]. The di-Boc-protected methyl glutamate was reduced to the corresponding aldehyde (**4**); subsequent Wittig olefination introduced the *E*-configured Michael acceptor moiety (**5**). Deprotection of the amino group and cleavage of the *tert*-butyl ester was followed by mono-Boc protection to yield building block **1**.

2-Hydroxynicotinic acid (**6**) was used as the starting material for the peptidomimetic building block (**2**). Initially, the carboxylic acid function was converted to the respective acyl azide by means of diphenylphosphoryl azide (DPPA), which subsequently underwent thermal decomposition (Curtius rearrangement) [39]. The isocyanate thereby formed was trapped with benzyl alcohol to generate the Cbz (benzyloxycarbonyl) protected amine (6.2, intermediate not shown in Figure 1). Alkylation with *tert*-butyl bromoacetate established the core pyridone (**7**). Cleavage of the *tert*-butyl ester and coupling with 2-ethylbutylamine resulted in the pyridone derivative (**8**), bearing the branched alkyl side-chain to address the hydrophobic cavity of TG2. Final hydrogenation to the corresponding aminopyridone yielded building block (**2**). Building blocks (**1**) and (**2**) were coupled to the Boc protected ZED1227 precursor (**9**). Deprotection of the amino group, followed by introducing 1-methylimidazole-5-carboxylic acid, finally yielded the active pharmaceutical ingredient ZED1227 as the free base. Supplementary information on the synthesis of ZED1227, including analytical data, can be found in Appendix A.

### 2.2. Physicochemical Features and Stability

The solubility of ZED1227 in phosphate-buffered saline (PBS, pH 7.4) and diluted HCl (pH 1.1) was determined by HPLC (Agilent 1260 Infinity, Santa Clara, CA, USA; column: Zorbax Eclipse XDB-C18, Agilent, Santa Clara, CA, USA, 1.8 μm, 4.6 × 50 mm^2^, gradient from 5 to 95% eluent B at 15%/min; eluent A: water, eluent B: ACN; 0.01% TFA; 40 °C; flow: 1.5 mL/min; detection: 214 nm). The logD value (distribution coefficient) of the compound was determined by means of the shake flask method [40,41], measuring the distribution of the solute in octanol and phosphate-buffered saline (PBS, pH 7.4). The concentration of ZED1227 in the immiscible solvents was determined by HPLC (*vide supra*). The stability in PBS (pH 7.4 or acidified to pH 5.0), in artificial gastric, and intestinal fluid [42,43] was calculated as the percentage of residual parent compound after incubation for 24 h at 37 °C, using an initial concentration of 100 μM.

### 2.3. Caco-2 Cell Permeability Assay

The permeability coefficient (P_app_ value) was obtained from Caco-2 monolayer studies predicting oral/intestinal bioavailability of the tested compound. The assay was performed using CacoReady^TM^ ready-to-use kits from ReadyCell according to the manufacturer’s protocol. Compounds with a P_app_ value below 1 × 10^−6^ cm/s are considered not to be permeable and therefore to have poor oral bioavailability. The concentration of ZED1227 in the basal and apical media was determined by HPLC. The initial concentration of ZED1227 in the apical medium was 200 μM. Propranolol (20 μM final concentration) was used as a control to validate each ready-to-use plate. Compound stock solutions were prepared in DMSO and further diluted to 1% DMSO using HBSS pH 6.5 to yield the desired working concentration.

### 2.4. Cytotoxicological Analysis

Cytotoxicological analysis of ZED1227 was performed using Huh7 (human liver carcinoma, CLS Cell Lines Service GmbH, Eppelheim, Germany) and Caco-2 cells (human epithelial colorectal adenocarcinoma, Sigma Aldrich, Taufkirchen, Germany) with respect to the effect on proliferation and viability, according to the assay protocols provided by the manufacturers of the respective kits. The Cell Proliferation ELISA (BrdU, colorimetric) was purchased from Roche (Mannheim, Germany), whereas the Cell Proliferation and Cytotoxicity Assay EZ4U was purchased from Biomedica (Vienna, Austria). Briefly, Caco-2 cells (in MEM with 1% non-essential amino acid solution, both Sigma Aldrich (Taufkirchen, Germany), 10% fetal bovine serum, 2 mM L-glutamine, 100 U/mL penicillin–streptomycin, all from Gibco) or Huh7 cells (in DMEM low glucose, Sigma Aldrich (Taufkirchen, Germany), 10% fetal bovine serum, 1 mM sodium pyruvate, 100 U/mL penicillin–streptomycin, all from Gibco) were cultured in 96 well plates, using 2.5 μg/mL cycloheximide and 0.2 μg/mL camptothecin-treated cells as positive control. In the 5-bromo-2′-deoxyuridine (BrdU) assay (determination of cell proliferation), the cells were incubated with ZED1227 working solution (1% DMSO in culture medium) up to a concentration of 1,000 μM for 24 h, followed by addition of BrdU into the culture medium and further culturing for 24 h. Detection of incorporated BrdU was performed through antibody binding and colorimetric detection.

The EZ4U assay (determination of viability) was performed by incubating the cells with ZED1227 working solution (1% DMSO in culture medium) up to a concentration of 1,000 μM for 48 h, followed by addition of a yellow tetrazolium reagent (not further specified) into the culture medium. The non-toxic tetrazolium salts were reduced to red-colored formazan which was quantified spectrophotometrically. As this reduction process required functional mitochondria, which are inactivated within a few minutes after cell death, this method provided an excellent tool for discrimination between living and dead cells.

### 2.5. Inhibition of Tissue Transglutaminase and Other Isoenzymes

Inhibition data were determined using the fluorescent transamidation assay (dansylcadaverine incorporation into glutamine-donor substrate *N*,*N*-dimethyl casein, DCC-assay) [44,45]. Briefly, to 15 μL of ZED1227 solution (dissolved and serial diluted in 2% (*v*/*v*) DMSO/assay buffer), 285 μL assay buffer [47.5 mM tris(hydroxymethyl)aminomethane, 9.5 mM CaCl_2_, 9.5 mM glutathione, 2.4% (*v*/*v*) glycerol, 190 mM NaCl, pH 8.0] containing dansylcadaverine (15.9 μM), *N*,*N*-dimethyl casein (3.8 μM) and the indicated recombinant transglutaminase enzyme [0.95 μg/mL hTG1 (T035), 0.81 μg/mL hTG2 (T022), 0.83 μg/mL hTG6 (T021), all from Zedira] were added, mixed and the kinetic measurement started immediately. In the case of hFXIII-A_2_ (0.83 μg/mL, T027, Zedira), the assay buffer contained human α-thrombin (5.2 × 10^−3^ U/μL, T056, Zedira). The mixture was incubated for 20 min at room temperature to activate FXIII. In the case of hTG3a (0.81 μg/mL, T013, Zedira), full enzymatic activity was obtained by incubation for 80 min at 37 °C in assay buffer before addition of the inhibitor and start of the kinetics.

Fluorescence emission was continuously monitored for 30 min at λ_em_ = 500 nm (λ_ex_ = 330 nm) and 37 °C using a CLARIOstar fluorescence plate reader (BMG Labtech, Ortenberg, Germany). All measurements were performed in triplicate. The respective IC_50_ (and IC_90_ for TG2) values were calculated by non-linear regression using the MARS software package (BMG Labtech). An irreversible-acting inhibitor, such as ZED1227, should, correctly, be characterized by the second-order rate constant k_2nd_, as detailed by Keillor [46]. However, such data are rather abstract and difficult to comprehend.

To facilitate comparability during lead structure optimization and to roughly estimate concentrations needed at the target site, the apparent IC_50_ values (calculated by four-parameter non-linear fit by MARS software) for irreversible inhibitors are more instructive and are therefore reported. Further, we report the potency of commercially available reference inhibitors Z006 (“Z-DON”), Z013 (ZED754), and T101 (all Zedira). To gain deeper mechanistic insight, we discuss the second-order rate constants (k_2nd_) published for Z006 and ZED754 in relation to their respective IC_50_ values.

### 2.6. Reactivity of ZED1227 towards Thiols

To exclude undesired reactions of ZED1227 with biological thiols, an investigation was performed with the tripeptide glutathione (GSH) as a surrogate. ZED1227 (60 μM) was incubated with a 160-fold excess of glutathione (10,000 μM) in aqueous media [containing 7% (*v*/*v*) tetrahydrofuran and 13% (*v*/*v*) methanol] at ambient temperature (25 °C) [47]. The parent compound and the GSH-adduct formed after 48 h were determined by HPLC (*vide supra*).

## 3. Results and Discussion

### 3.1. Inhibitor Design

The goal of the drug-discovery project was to provide a potent and selective tissue transglutaminase inhibitor as a potential drug candidate for the treatment of celiac disease. Several considerations come into play when designing efficacious human tissue transglutaminase inhibitors. The published X-ray co-crystal structure [48] with an inhibitor implied only shallow pockets for recognition of substrates (inhibitors), representing a significant impediment in the drug-discovery project. Covalent adduct formation is an attractive approach to address challenging targets and to improve the potency of inhibitors. As an example, the human rhinovirus 3C protease blocker AG7088 (Rupintrivir, Agouron Pharmaceuticals) was developed to treat the common cold [49]. While the peptidomimetic backbone provides the necessary affinity for the viral protease, a C-terminal Michael acceptor warhead irreversibly forms a covalent adduct with the active site cysteine.

**Figure 2:**
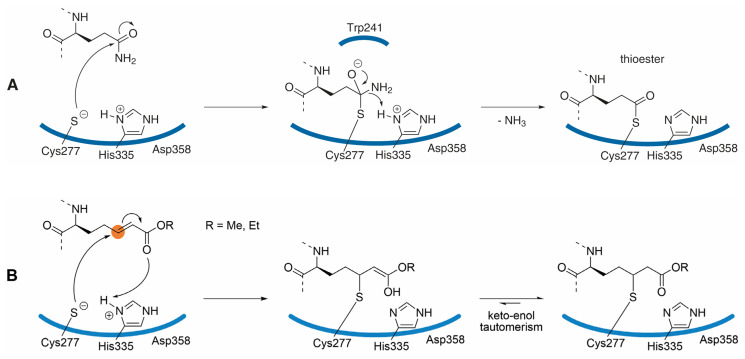
(**A**) Transglutaminase reaction mechanism: the catalytic triad of human tissue transglutaminase is formed by the amino acids Cys277-His335-Asp358. The proposed thiolate-imidazolium ion pair is exceptionally nucleophilic enabling the attack of the otherwise inert carboxamide side-chain of protein bound glutamine to yield the thioester intermediate that itself is prone to react with the ε-amino group of lysine (not shown). The proposed tetrahedral oxyanion is stabilized by Trp241 and by the backbone nitrogen of Cys277; the driving force of the reaction is the release of ammonia. Hydrolysis of the reactive thioester is suppressed by the narrow hydrophobic tunnel excluding water from the catalytic site [48]. (**B**) The Michael acceptor warhead mimics the substrate glutamine side-chain, and when embedded in a suitable peptidic/peptidomimetic backbone the warhead addresses the catalytic center of tissue transglutaminase. The cysteinyl γ thiolate moiety of Cys277 attacks the complementary electrophilic β-carbon (marked in orange) of the α,β-unsaturated ester. The Michael addition to the alkene leads to the covalent, irreversible inhibition of TG2, following the mechanism previously described for cysteine proteases [51].

**Figure 3:**
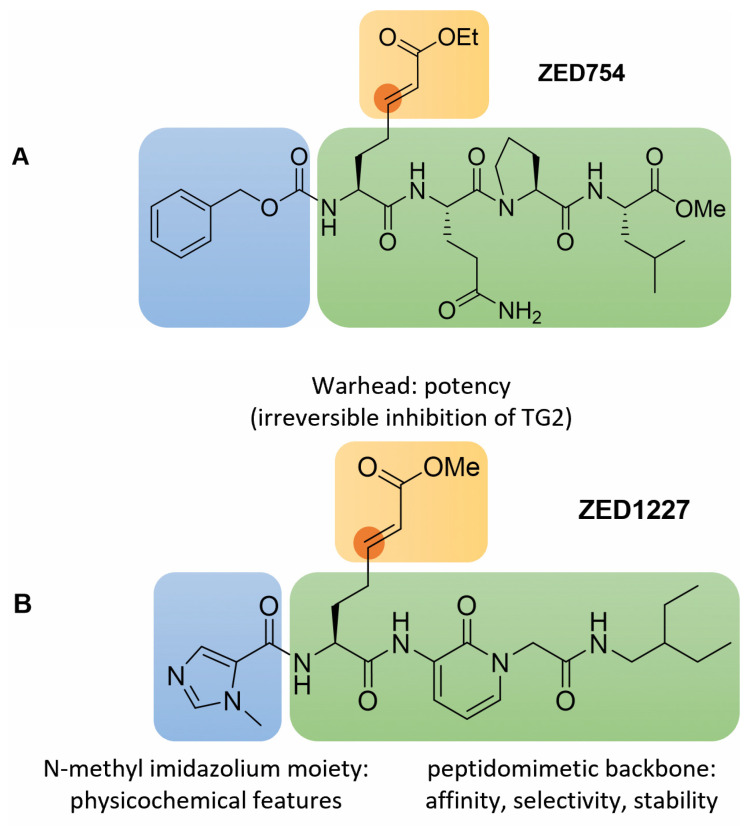
(**A**) The peptide lead structure ZED754 bears the N-terminal Cbz (benzyloxycarbonyl) protecting group (blue) followed by an α,β-unsaturated ethyl ester warhead (yellow; the electrophilic β-carbon is marked in orange) attached to a tripeptide, Gln-Pro-Leu-OMe (green). ZED754 already provides the blueprint for the overall chemical architecture of the drug candidate ZED1227 as illustrated by the color code. (**B**) The structural features of ZED1227 are highlighted in color as follows: The peptidomimetic backbone (green) provides affinity to the target guiding the Michael acceptor warhead (yellow) into the catalytic center of active tissue transglutaminase. The electrophilic β-carbon (marked in orange) of the *E*-configured α,β-unsaturated methyl ester is attacked with high efficiency by the uniquely nucleophilic thiolate moiety of Cys277. The N-terminal N-methyl imidazolium heterocycle (blue) is a weak base and thereby contributes to the overall physicochemical features of ZED1227, especially the pH dependent solubility profile.

In contrast to this virus protease blocker, when using the Michael acceptor warhead in transglutaminase inhibitors, the electrophilic β-carbon (marked in orange, Figure 2B) of the α,β-unsaturated ester takes the place of the γ-carboxamide of the substrate glutamine side-chain (Figure 2A). The proposed mechanism of transglutaminase inhibition, yielding a stable thioether, is outlined in Figure 2B. This mode of inhibition is supported by the X-ray co-crystal structure of TG2 with ZED754 (*vide infra*); ZED754 may be considered as a peptidic lead structure for the drug candidate ZED1227 already containing the Michael acceptor warhead (see Figure 3). As a comparison, the classical transglutaminase transamidation pathway proceeds via a reactive thioester intermediate (Figure 2A) that is prone to nucleophilic attack.

The catalytic center of TG2 shows some similarity to that of cysteine proteases, especially the fact that the active site thiol of Cys277 is exceptionally nucleophilic due to interaction (charge transfer) with His335 as part of the catalytic triad, forming a thiolate-imidazolium ion pair analogous to that described in FXIII-A_2_ [50]. Importantly, the intrinsic reactivity of the Michael acceptor warhead is too low to drive undesired reactions with biological thiols, such as glutathione.

### 3.2. Structural Features and In Vitro Profiling of ZED1227

The chemical structure of ZED1227 with highlighted relevant structural features is displayed in Figure 3B. The peptidomimetic backbone (green) of the synthetic compound (molecular mass 528.6 g/mol) provides the necessary affinity for the target, guiding the Michael acceptor warhead (yellow) into the catalytic center of tissue transglutaminase. Overall, the structure of ZED1227 has strong similarity to a preferred peptide substrate, Cbz-QQPL-OMe, that was identified in a screening, whereby “Cbz” represents the N-terminal benzyloxycarbonyl protecting group of the tetrapeptide (one letter code). Replacing the highlighted glutamine (Q), which serves as the primary acyl-donor in the transglutaminase reaction, by a Michael acceptor warhead, led to the ZED754 compound (Figure 3A). ZED754 was co-crystallized with recombinant human tissue transglutaminase, essentially following the published procedure [48]; The X-ray co-crystal structure was deposited in the Protein Data Bank in 2012 as PDB-ID: 3S3P (Lindemann et al.). The formation of the covalent thioether adduct was resolved in this structure. As peptides are generally not preferred as active pharmaceutical ingredients, they are typically replaced by artificial building blocks. In line with this notion, the glutamine-proline dipeptide was substituted by the aminopyridone-acetyl structure. As a note, very recently, the aminopyridone motive, hiding a former peptide bond, was successfully implemented in a drug discovery project addressing the main protease of severe acute respiratory syndrome–coronavirus 2 (SARS-CoV-2) [52]. Further, the ethylbutylamine moiety was chosen as a mimic of the branched leucine alkyl group in the ZED754 lead structure. Detailed discussion of the lead optimization program is beyond the scope of the present article and will be reported elsewhere.

Of particular interest for the overall physicochemical properties of the compound is the N-terminal ligand (blue, Figure 3B). The N-methyl imidazolium heterocycle is a weak base and, as such, is protonated at low pH. This feature underpins the excellent solubility of the ZED1227 compound in the stomach (25.9 g/L at pH 1.1). In contrast, the upper part of the small intestine (duodenum), which is the target tissue, is slightly acidic to neutral, and the solubility of ZED1227 at pH 7.4 is rather low (0.05 g/L). Furthermore, detailed analysis in different fluidic environments showed that ZED1227 is most stable at low pH as well as in simulated gastric and intestinal fluid (recovery >80% after 24 h). The stability at physiological pH 7.4 is remarkably lower (10% recovery of the parent compound after 24 h incubation at 37 °C). This instability is caused by intramolecular cyclization with consequential inactivation of the warhead. The Aza–Michael reaction yields both the five- and six-membered ring derivative, depending on the attacking nitrogen within the peptidomimetic backbone. The logD value (distribution coefficient in octanol and phosphate-buffered saline), on the other hand, was determined as 2.0, indicating that the lipophilicity falls well into the generally acceptable window for drugs. Despite the fact that various irreversible inhibitors have been approved as drugs, shown to be safe, and commercially successful, this has not completely overcome the fear of potential drug-induced toxicity [53]. Accordingly, studies to exclude off-target reactions with biologically relevant thiols are needed. As a step in this direction, a study with ZED1227 in the presence of glutathione (160-fold excess) yielded only 2% glutathione-adduct, while recovering 98% of the parent compound after 48 h. Most notably, ZED1227 was not cytotoxic up to 1,000 μM concentration when added to cultures of Huh7 and Caco-2 cells, nor did it affect cell proliferation rates. Table 1 summarizes some key features of the candidate drug.

TG2 is ubiquitously expressed throughout the human body and has pleiotropic functions. Therefore, it was important to consider potentially unintended consequences besides the desired pharmacodynamic efficiency in inhibition of tissue transglutaminase activity in the extracellular space of the inflamed gut. Tissue transglutaminase is most abundant intracellularly in a catalytically inactive state due to low calcium/high GTP concentration. ZED1227, as a mechanism-based inhibitor, is very unlikely to react with the inactive tissue transglutaminase. Furthermore, cell permeability of the compound is low (less than 1 × 10^−6^ cm/s) as determined by the Caco-2 assay. Therefore, it is unlikely that the transglutaminase inhibitor could affect the various functions assigned to TG2 in intact cells, at least under normal physiological conditions. Most notably, TG2-knockout mice have normal embryonic and post-natal development and are phenotypically healthy, at least under stress-free conditions [54], suggesting that TG2 is likely to be a safe therapeutic target for drug development.

### 3.3. Potency and Selectivity of ZED1227

The ZED1227 compound is a very potent inhibitor of human tissue transglutaminase (IC_50_ = 53 nM; IC_90_ = 300 nM) and was profiled here against the human transglutaminase isoenzymes considered to be the major off-targets (FXIII, TG1, TG3, and TG6). The same transamidation assay was used as all transglutaminases have been shown to accept *N,N*-dimethyl casein as glutamine-donor substrate [44,45]. As shown in Table 2, ZED1227 proved to have excellent selectivity (122-fold for TG6; up to >900-fold for TG3). Special attention was paid here to coagulation factor XIII (FXIII, F13), also called plasma transglutaminase. FXIII represents the last enzyme in the coagulation cascade and is responsible for crosslinking and stabilization of fibrin fibers. FXIII plays a key role in clot formation, maturation, and composition. Congenital FXIII deficiency can be associated with bleeding tendency, though severity differs between individuals affected. The selectivity of ZED1227 towards recombinant FXIII-A_2_ (catalytic active A-subunit) in the transamidation assay (IC_50_ = > 50,000 nM; selectivity of >900-fold) was found to be excellent. Hence, even if ZED1227 shows some systemic exposure, inhibition of FXIII can be virtually excluded.

There are quite a lot of different transglutaminase assay systems and settings used in different laboratories. This makes it virtually impossible to compare the potency of different transglutaminase inhibitors based on the data reported. To facilitate comparability, we also report here the potency of three frequently used commercially available reference inhibitors in the same standardized assay. ZED754, the peptidic lead structure to ZED1227 (Figure 3), is about 3.5-fold less potent (IC_50_ = 182 nM). The IC_50_ determined for Z006 (“Z-DON”, Z-DON-Val-Pro-Leu-OMe, Zedira) was 70 nM, which is only slightly lower than the potency of ZED1227 (IC_50_ = 53 nM) despite the intrinsic reactivity of the warhead of Z006 being higher. The pan-transglutaminase oxopropyl thioimidazolium inhibitor T101 (L-682.777, Zedira), originally developed as an FXIIIa blocker by Merck Sharp & Dohme, was 33-fold less potent (IC_50_ = 1,780 nM) compared to ZED1227 in the same experimental setting.

The literature provides additional data for ZED754 (k_2nd_ = 51,500 M^−1^s^−1^, K_i_ = 0.45 μM) and Z006 (k_2nd_ = 191,000 M^−1^s^−1^, K_i_ = 0.096 μM) derived from kinetic measurement of the TG2 catalyzed hydrolysis of the small molecule Z-Glu(HMC)-Gly-OH at pH 6.5 [55]. Considering the similar potency of Z006 and ZED1227, we assume the second-order rate constant of ZED1227 to be at least in the same range. This assumption is supported by the k_2nd_ value, and the X-ray structure reported for ZED754 favoring the usage of the Michael-acceptor warhead for the inhibition of (tissue) transglutaminase. In conclusion, the above outlined features of ZED1227 qualified the new chemical entity as a drug candidate ready to enter regulatory preclinical and clinical development.

### 3.4. Early Clinical Development

The phase 1 clinical trial involved more than 100 healthy individuals exposed to up to 500 mg of ZED1227. The ZED1227 compound proved to be safe and well-tolerated in healthy female and male volunteers (EudraCT numbers, 2014-003044-13 and 2015-005283-42). Accordingly, the new chemical entity was progressed to a phase 2a clinical study. The efficacy and safety of a 6-week treatment with ZED1227 was assessed in a randomized, double-blind, placebo-controlled, dose-finding trial at three dose levels (10, 50, 100 mg). In this trial, adult patients with well-controlled celiac disease were challenged with 3 g daily gluten intake to assess the protective effect of ZED1227 on the mucosa. The encouraging clinical data were recently published [36], with the key finding that all dose levels attenuated gluten-induced duodenal mucosal injury. Based on the promising results a phase 2b study was initiated in celiac disease patients suffering from symptoms despite following a “gluten-free diet”—essentially an adjunct treatment. There will be no gluten challenge in this “real-life” study, which aims to further substantiate the protective effect of ZED1227 and impact on quality of life in celiac disease patients. It remains to be seen whether the promising clinical data obtained so far translate into a viable, commercially successful drug for patient benefit.

## 4. Conclusions and Future Perspective

Here, we described key features of ZED1227, the first tissue transglutaminase (TG2) inhibitor in clinical trials. ZED1227 harbors a peptidomimetic backbone that underpins the high affinity for TG2. However, the target surface on tissue transglutaminase is rather shallow with no addressable deep-binding pockets which has hampered the development of potent compounds. This impediment was overcome by introducing an electrophilic warhead that irreversibly blocks the enzyme, while at the same time substantially increasing the potency. While a “resurgence” of such suicide inhibitors is recognized to a certain extent [56], reluctance is still prevalent within the pharmaceutical industry due to potential drug-induced injury. Another general concern is the potential side-effects associated with the drug target itself. TG2 is expressed throughout the human body in virtually all tissues and organs. Even if the physiological functions of tissue transglutaminase remain mostly elusive, the fear of adverse events when blocking TG2 activity is salient. Further, TG2 is a member of the transglutaminase family consisting of eight active isoenzymes. Accordingly, selectivity of a drug candidate is key to avoid side-effects. Most importantly, coagulation factor XIII needs to remain unaffected due to the risk of compromised blood clot formation and a bleeding tendency. In summary, there have been a number of theoretical and practical obstacles that have had to be overcome to advance this drug discovery project to the clinical stage.

Some of the impediments could be conceptually addressed, through optimizing the features of the ZED1227 compound, especially the solubility profile, potency, and selectivity. The methyl imidazolium heterocycle facilitates solubility at low pH, as found in the stomach. Proportional to the increase in the pH value along the gut, the solubility of ZED1227 decreases reciprocally. Even if the compound might tend to precipitate at the site of the target tissue—the upper small intestine (duodenum)—there will be sufficient drug dissolved to achieve efficacy; for example, 100 μM at pH 7.4, exceeding the IC_90_ (300 nM) by a factor of 330. Notably, the IC_90_ corresponds to the concentration needed to block 90% of TG2 activity in a certain assay system and experimental setting. We used the classical casein [3.8 μM]/dansylcadaverine [15.9 μM] transamidation assay containing TG2 at 10 nM concentration. This assay/setting is the most challenging one of our portfolio elaborated on here. Even if it is not entirely clear how the artificial in vitro transamidation assay translates to the in vivo situation, we would assume a quantitative inhibition of active TG2 that is accessible. Further, the insoluble compound may not be bioavailable, lowering potential systemic exposure. The local (upper intestine), topical (extracellular, luminal surface), non-systemic drug-design concept is further manifest through the low cellular permeability of ZED1227. Another important feature increasing the safety margin of a compound is the mode-of-inhibition. Basically, ZED1227 inactivates only catalytically active, extracellular TG2 whereas the vast majority of the enzyme is trapped in its silent GTP-bound conformation within cells.

The backbone provides non-covalent recognition to ZED1227 and guides the warhead into the catalytic center of active tissue transglutaminase. The peptidomimetic design provides both affinity and selectivity to ZED1227. The carefully tuned warhead is well-orientated to react irreversibly with the highly nucleophilic cysteine as part of the catalytic triad, but not with biological thiols, such as glutathione.

In conclusion, ZED1227 is a very potent and selective inhibitor of tissue transglutaminase. The compound has fulfilled all regulatory pre-clinical requirements to enter human trials. The phase 1 study involved more than 100 healthy volunteers, and the phase 2a clinical trial assigned 123 patients to the three verum groups. Overall, the compound was shown to be safe and well-tolerated.

Of outmost importance for a first-in-class compound, besides safety, is efficacy. The proof-of-concept study included a 3 g daily gluten challenge in celiac patients. All three doses of ZED1227 assessed (10, 50, and 100 mg, oral dosage), protected celiac patients from mucosal damage caused by inflammation. The results imply that the daily dose required for the treatment of celiac disease, with the aim of protection from rather small amounts of hidden gluten in support of the gluten-free diet, might be well below 100 mg of ZED1227. This data must now be substantiated and expanded upon in the current phase 2b “real-life” study and in pivotal studies to follow. Remarkably, ZED1227 has clinically validated human tissue transglutaminase as a druggable target for the first time.

The study results not only provide hope to celiac disease patients for a safe and efficacious drug in the foreseeable future, but the impact of these findings extends far beyond celiac disease. The therapeutic approach of transglutaminase inhibition might translate to other indications, especially to fibrotic disorders, for which there is still a strong unmet medical need for alternative treatment options. Addressing this brings new challenges. New compounds must be designed to reach the circulation and eventually the organs affected by tissue fibrosis. Safety concerns will remain, or even be exacerbated, when considering the targeting of systemic disorders. There will certainly be the need for development of novel potent and selective reversible-acting inhibitors of tissue transglutaminase, as well as modulators of distinct TG2 functions other than its enzymatic activity.

Our findings have given new impetus to a long-neglected and widely overlooked enzyme family by the pharmaceutical industry. It is obvious that celiac disease has shone the spotlight on transglutaminases, accelerating future research and development in both academia and industry. Other compounds and antibodies addressing active TG2 are already in clinical development (e.g., Zampilimab by UCB/Chiesi and GSK3915393 by GlaxoSmithKline), and presumably more projects will follow, as is the usual practice in the pharmaceutical industry after clinical proof-of-concept of a new pharmaceutical approach has been demonstrated.

We also need to invest in more extensive efforts to obtain a better understanding of the biochemistry and distinct functions of other members of the transglutaminase family, such as TG6 or TG3. These enzymes are also involved in gluten-related disorders and are therefore potential drug targets. Furthermore, the development of inhibitors of coagulation factor XIII may prove to be clinically valuable as it constitutes a widely untapped target in anticoagulation. In conclusion, the decade for translation of research findings in the transglutaminase field to application is yet to come for academia, and for the pharmaceutical and diagnostic industries.

## Figures and Tables

**Table 1:** In vitro profiling of ZED1227.

Molecular Mass [g/mol]	528.6
Solubility at pH 7.4 Solubility at pH 1.1	100 μM/0.05 g/L 48,900 μM/25.9 g/L
LogD (octanol/PBS pH 7.4)	2.0
Stability in PBS/saline (24 h, 37 °C)	10% at pH 7.4 89% at pH 5.0
Stability in artificial gastric and intestinal fluid (24 h, 37 °C)	95%, simulated gastric fluid (pH 1.2) 82%, simulated intestinal fluid (pH 6.5)
A-B permeability (Caco-2-assay)	P_app_ < 1 × 10^−6^ cm/s
Cytotoxicity Proliferation: Caco-2 cells Huh7 cells Viability: Caco-2 cells Huh7 cells	no effect up to 1,000 μM no effect up to 1,000 μM no effect up to 1,000 μM no effect up to 1,000 μM
Reactivity towards excess glutathione (25 °C)	98% parent drug compound recovery (after 48 h)

**Table 2:** Potency and selectivity of ZED1227 against recombinant human transglutaminase isoenzymes.

ZED1227	hTG2	hTG1	hTG3	hTG6	hFXIII-A_2_
**App. IC_50_**	53 nM	24,863 nM	>50,000 nM	6,441 nM	>50,000 nM
**Selectivity**		469	>900	122	>900

## Abbreviations

aa: amino acid; TG2: tissue transglutaminase; ECM: extracellular matrix; GTP: guanosine triphosphate; GDP: guanosine diphosphate; DGPs: deamidated gliadin peptides; APCs: antigen-presenting cells; FXIII-A_2_: blood coagulation factor XIII A-subunit (homodimer), plasma transglutaminase; DON: diazo-5-oxonorleucine; PBS: phosphate-buffered saline; HPLC: high-performance liquid chromatography; logD: distribution coefficient; P_app_: permeability coefficient; DMSO: dimethyl sulfoxide; hTG1: human keratinocyte transglutaminase; hTG6: human neuronal transglutaminase; hTG3a: human epidermal transglutaminase, activated; IC_50/90_: inhibitor concentration for 50%/90% inhibition; GSH: glutathione; HBSS: Hanks’ balanced salt solution; MEM: minimum essential media; DMEM: Dulbecco’s Modified Eagle Medium; Boc: *tert*-butoxycarbonyl protecting group; Cbz: benzyloxycarbonyl protecting group; HMC: 7-hydroxy-4-methylcoumarin.

## Appendix A

### Appendix A.1. Supplementary Abbreviations

ESI-MS: electrospray ionization mass spectrometry; NMR: nuclear magnetic resonance spectroscopy; DMF: dimethylformamide; Boc_2_O: di-*tert*-butyl decarbonate; DMAP: 4-dimethylaminopyridine; ACN: acetonitrile; DIBAL-H: diisobutylaluminium hydride; Ph_3_P=CH-CO_2_Me: (carbomethoxymethylene)triphenylphosphorane; TFA: trifluoroacetic acid; DCM: dichloromethane; Boc-OSu: N-(*tert*-butoxycarbonyloxy)succinimide; DIPEA: *N*,*N*-diisopropylethylamine; DPPA: diphenylphosphoryl azide; TEA: triethylamine; THF: tetrahydrofuran; TBTU: 2-(1H-Benzotriazole-1-yl)-1,1,3,3-tetramethylaminium tetrafluoroborate; HOBt: 1-hydroxybenzotriazole; Pd/C: palladium on activated charcoal; HATU: (1-[bis(dimethylamino)methylene]-1H-1,2,3-triazolo[4,5-b]pyridinium 3-oxide hexafluorophosphate.

### Appendix A.2. Synthesis

All chemicals, reagents and solvents were purchased from commercial sources and used without additional purification. ESI-MS spectra were acquired on a Shimadzu LCMS-2020 single quadrupole mass spectrometer. NMR spectra were recorded on a Bruker Avance 500 MHz NMR instrument.

(***S***)**-1-*tert*-Butyl 5-methyl 2-**(***tert*-butoxycarbonylamino**)**pentanedioate** (**3.1**, intermediate not shown in Figure 1). An amount of 12.0 g of Boc-Glu-OtBu (39.6 mmol) was dissolved in 200 mL of DMF. Under argon atmosphere, 7.09 g of cesium carbonate (21.8 mmol, 0.55 eq.) was added and the resulting suspension was stirred for 1 h at room temperature (RT). Subsequently, 2.47 mL of methyl iodide (39.6 mmol) was added and stirred at RT overnight. The solvent was removed in vacuo and the obtained residue was taken up in 400 mL of ethyl acetate. The precipitate was removed by filtration and the filtrate was washed 3 times with each of 75 mL of 10% citric acid, 10% NaHCO_3_ solution and brine (NaCl solution, concentrated). The organic phase was dried over Na_2_SO_4_, filtered, and the solvent was evaporated in vacuo. The product was obtained as yellow oil (13.4 g, >100%, ESI-MS: 340.2 [M + Na]^+^) and used without further purification.

(***S***)**-1-*tert*-Butyl 5-methyl 2-**(**bis**(***tert*-butoxycarbonyl**)**amino**)**pentanedioate** (**3.2**, intermediate not shown in in Figure 1). An amount of 13.4 g of Boc-Glu(OMe)-OtBu (**3.1**, ~39.6 mmol) was dissolved in 30 mL of acetonitrile and treated with 986 mg of DMAP (7.91 mmol, 0.2 eq). Under nitrogen atmosphere, a solution of 17.6 g of di-*tert*-butylbicarbonate (77.1 mmol, 2 eq) in 100 mL of acetonitrile was added. After stirring overnight, the solvent was removed in vacuo and the obtained crude product was purified by chromatography on silica gel 60 (0.04–0.063 mm, column: 31 × 6 cm^2^, petroleum ether/ethyl acetate 9:1, collected in 250 mL fractions, product: fractions 6–13, thin-layer chromatography (TLC control): petroleum ether/ethyl acetate 8:2, R_f_ = 0.70). The product was obtained as a pale yellow oil (13.7 g, 32.8 mmol, 83%, ESI-MS: 440.3 [M + Na]^+^).

(***S***)**-*tert*-Butyl 2-**(**bis**(***tert*-butoxycarbonyl**)**amino**)**-5-oxopentanoate** (**4**). An amount of 13.7 g of Boc_2_-Glu(OMe)-OtBu (**3.2**, 32.8 mmol) was dissolved in 200 mL of absolute diethylether and cooled to −78 °C under argon atmosphere. At this temperature, 36.1 mL (36.1 mmol, 1.1 eq) of a solution of diisobutyl aluminum hydride (1 M in hexane) was added dropwise. After the addition, the solution was stirred for a further 15 min at −78 °C, before the reaction was quenched by addition of 50 mL of water. With vigorous stirring, the suspension was warmed to RT and filtered over Celite^®^. The filtrate was concentrated, and the residual water was removed by azeotropic distillation with toluene. The product was obtained as pale yellow oil (13.3 g, >100%, ESI-MS: 410.4 [M + Na]^+^) and used without further purification.

(***S*,*E***)**-7-*tert*-Butyl 1-methyl 6-**(**bis**(***tert*-butoxycarbonyl**)**amino**)**hept-2-enedioate** (**5**). An amount of 13.2 g of Boc_2_-Glu(H)-OtBu (**4**, ~32.8 mmol) was dissolved in 20 mL of dried benzene. Under argon atmosphere, 11.2 g of (methoxycarbonylmethylen)-triphenyl-phosphorane (32.8 mmol) was added portion-wise at RT. After stirring overnight, the solvent was removed in vacuo and the oily residue was purified by chromatography on silica gel 60 (0.04–0.063 mm, column: 39 × 6 cm^2^, petroleum ether/ethyl acetate 9:1, collected in 250 mL fractions, product: fractions 2–12, TLC: petroleum ether/ethyl acetate 8:2, R_f_ = 0.54). The product was obtained as a pale yellow oil (12.0 g, 27.1 mmol, 83%, ESI-MS: 466.3 [M + Na]^+^).

(***S*,*E***)**-2-**(***tert*-Butoxycarbonylamino**)**-7-methoxy-7-oxohept-5-enoic acid** (**1**). 7.0 g of (*S*,*E*)-7-*tert*-butyl 1-methyl 6-(bis(*tert*-butoxycarbonyl)amino)hept-2-enedioate (**5**, 15.8 mmol) was dissolved in 40 mL of DCM before 70 mL of TFA were added dropwise. The solution was stirred for 4 h at RT before the solvent was removed in vacuo. The oil was dissolved in 50 mL of DMF. By successive addition of DIPEA, the pH value of the solution was adjusted to 6–7. Subsequently, 4.08 g of Boc-OSu (18.9 mmol, 1.2 eq) and 5.37 mL of DIPEA were added and the reaction was stirred overnight at RT. The solvent was removed in vacuo and the residue was suspended in 130 mL of 5% KHSO_4_ solution. The slurry was extracted with ethyl acetate (1 × 150 mL, 2 × 100 mL) and the combined organic phases were washed with brine (75 mL). The organic phase was dried over Na_2_SO_4_, filtered, and the solvent was evaporated in vacuo. The residue was purified by chromatography on silica gel 60 (0.04–0.063 mm, column: 13 × 6 cm^2^, toluene/ethyl acetate 65:35, 0.5% acetic acid, collected in 200 mL fractions, product: fractions 2–5, TLC: toluene/ethyl acetate 1:1, 0.5% acetic acid, R_f_ = 0.35). The product was obtained as colorless oil (4.04 g, 89%, ESI-MS: 310.1 [M + Na]^+^).

**Benzyl N-**(**2-hydroxypyridin-3-yl**)**carbamate** (**6.2**, intermediate not shown in in Figure 1). An amount of 15 g of 2-hydroxy-nicotinic acid (108 mmol) was suspended in 180 mL of dried dioxane. The suspension was cleared by addition of 14.9 mL of triethylamine (108 mmol). A quantity of 24 mL of DPPA (108 mmol) was added and the reaction solution was refluxed (130 °C) under argon atmosphere. After 16 h, a further 16.3 mL of TEA and 12.8 mL of benzyl alcohol (117 mmol, 1.1 eq) were added successively and refluxed for another 24 h. The solvent was removed in vacuo and the obtained brown residue was taken up in 600 mL of DCM/brine (1:1). The solution was adjusted to 1 by addition of about 22 mL of 1M HCl. The aqueous phase was extracted with DCM (2 × 200 mL) and the combined organic phases were washed with 10% NaHCO_3_ solution (3 × 150 mL) and brine (1 × 150 mL), dried over Na_2_SO_4_, filtered, and the solvent was evaporated in vacuo. The brown solid obtained was recrystallized from 300 mL of methanol to yield a pale brown solid (16.2 g, 62%, ESI-MS: 245.1 [M + H]^+^).

***tert*-Butyl 2-**(**3-**(**benzyloxycarbonylamino**)**-2-oxopyridin-1**(**2H**)**-yl**)**acetate** (**7**). An amount of 16.2 g of benzyl-3-hydroxypyridin-3-yl-carbamate (**6.2**, 66.4 mmol) was suspended in 900 mL of absolute THF and cooled to 0 °C under argon atmosphere. An amount of 2.92 g of NaH (60% in mineral oil, 73.1 mmol, 1.1 eq) was added portion-wise. After the end of gas emission (15 min), 13.7 mL of bromoacetic acid *tert*-butylester (89.7 mmol, 1.35 eq) was added to the solution. The reaction was stirred at 0 °C for 15 min and for a further 16 h at RT. The mixture was filtered, and the filtrate was concentrated to dryness. The residue was taken up in 5 mL of ethyl acetate and then treated with 50 mL of diethylether. The resulting suspension was precipitated in the refrigerator overnight. The crystals were collected by filtration and washed with cold ether (19.3 g, 81%, ESI-MS: 359.1 [M + H]^+^).

**2-**(**3-**(**Benzyloxycarbonylamino**)**-2-oxopyridin-1**(**2H**)**-yl**)**acetic acid** (**7.1**, intermediate not shown in in Figure 1). An amount of 4.00 g of *tert*-butyl 2-(3-(benzyloxycarbonylamino)-2-oxopyridin-1(2H)-yl) acetate (**7**, 11.2 mmol) was dissolved in 50 mL of DCM before 50 mL of TFA was added dropwise. The solution was stirred for 3 h at RT before the solvent was removed in vacuo to yield a brown solid (3.70 g, >100%, ESI-MS: 303.2 [M + H]^+^) which was used without further purification.

**Benzyl-1-**(**2-**(**2-ethylbutylamino**)**-2-oxoethyl**)**-2-oxo-1,2-dihydropyridin-3-ylcarbamate** (**8**). A mixture of 3.70 g of 2-(3-(benzyloxycarbonylamino)-2-oxopyridin-1(2H)-yl)acetic acid (**7.1**, ~11.2 mmol), 3.58 g of TBTU (11.2 mmol) and 1.51 g of HOBt (11.2 mmol) was dissolved in 60 mL of DMF. The pH value was adjusted to 9–10 by addition of 5.70 mL of DIPEA (33.5 mmol, 3 eq). An amount of 1.50 mL of 2-ethyl-butylamine (11.2 mmol) was added and the mixture was stirred at RT overnight. The solvent was removed in vacuo and the obtained residue was taken up in 300 mL of DCM and subsequently washed with 10% citric acid (3 × 75 mL), saturated NaHCO_3_ solution (3 × 75 mL) and brine (75 mL). The organic phase was dried over Na_2_SO_4_, filtered, and the solvent was evaporated in vacuo. Pale brown solid (5.22 g, >100%, ESI-MS: 386.3 [M + H]^+^) was obtained and used without further purification.

**2-**(**3-Amino-2-oxopyridin-1**(**2H**)**-yl**)**-N-**(**2-ethylbutyl**)**acetamide** (**2**). An amount of 5.22 g of benzyl-1-(2-(2-ethylbutylamino)-2-oxoethyl)-2-oxo-1,2-dihydropyridin-3-ylcarbamate (**8**, ~11.2 mmol) was dissolved in 60 mL of methanol under nitrogen atmosphere. To this solution, 500 mg of Pd/C (10%) was added and stirred for 2.5 h under hydrogen atmosphere at atmosphere pressure. The mixture was filtered over silica gel, before the solvent was removed in vacuo. A green to brown solid (3.62 g, >100%, ESI-MS: 252.2 [M + H]^+^) was obtained and used without further purification.

**(*S*,*E*)-Methyl 6-(*tert*-butoxycarbonylamino)-7-(1-(2-(2-ethylbutylamino)-2-oxoethyl)-2-oxo-1,2-dihydropyridin-3-ylamino)-7-oxohept-2-enoate** (**9**). A solution of 3.36 g of 2-(3-amino-2-oxopyridin-1(2H)-yl)-N-(2-ethylbutyl)acetamide (**2**, ~10.4 mmol) in 20 mL of DMF was provided. To this solution, a solution of 2.97 g of (*S*,*E*)-2-(*tert*-butoxycarbonylamino)-7-ethoxy-7-oxohept-5-enoic acid (**1**, 10.4 mmol), 3.93 g of HATU (10.4 mmol) and 3.52 mL of DIPEA (20.7 mmol, 2 eq) in 40 mL of DMF was added. By successive addition of DIPEA, the pH value was adjusted to 7–8. The reaction mixture was stirred at 40 °C overnight, before the solvent was removed in vacuo. The obtained brown residue was taken up in 250 mL of ethyl acetate and subsequently washed with 10% citric acid (3 × 75 mL), saturated NaHCO_3_ solution (3 × 75 mL) and brine (75 mL). The organic phase was dried over Na_2_SO_4_, filtered, and the solvent was evaporated in vacuo. The residue was purified by chromatography on silica gel 60 (0.04–0.063 mm, column: 13 × 6 cm^2^, toluene/acetone 7:3, collected in 40 mL fractions, product: fractions 6–15, TLC: DCM/MeOH = 97/3, Rf = 0.40) to yield a pale brown solid (3.34 g, 62%, ESI-MS: 543.4 [M + Na]^+^),

**(*S*,*E*****)-Methyl 7-(1-(2-(2-ethylbutylamino)-2-oxoethyl)-2-oxo-1,2-dihydropyridin-3- ylamino)-6-(1-methyl-1H-imidazole-5-carboxamido)-7-oxohept-2-enoate** (**ZED1227**). An amount of 3.14 g of (*S*,*E*)-methyl 6-(*tert*-butoxycarbonylamino)-7-(1-(2-(2-ethylbutylamino)-2-oxoethyl)-2-oxo-1,2-dihydropyridin-3-ylamino)-7-oxohept-2-enoate (**9**, 6.03 mmol) was dissolved in 25 mL of DCM before 35 mL of TFA was added dropwise. The solution was stirred for 3 h at RT before the solvent was removed in vacuo. The obtained brown oil was dissolved in 10 mL of DMF and 1.03 mL of DIPEA (6.03 mmol) was added. A solution of 760 mg of 1-methyl-1H-imidazole-5-carboxylic acid (6.03 mmol), 2.29 g of HATU (6.03 mmol) and 1.03 mL of DIPEA (6.03 mmol) in 30 mL of DMF was added. By successive addition of DIPEA the pH value was adjusted to 7–8 and the reaction was stirred at RT overnight before the solvent was removed in vacuo. The residue was taken up in 200 mL of ethyl acetate and subsequently washed with 10% citric acid, saturated NaHCO_3_ solution and brine (each 75 mL). The organic phase was dried over Na_2_SO_4_, filtered, and the solvent was evaporated in vacuo. The residue was purified by chromatography on silica gel 60 (0.04–0.063 mm, column: 12 × 6 cm^2^, DCM/MeOH 95:5, collected in 50 mL fractions, product: fractions 43–66, TLC: DCM/MeOH 97:3, R_f_ = 0.30) to yield ZED1227 as a pale brown solid (1.42 g, 45%, ESI-MS: 529.37 [M + H]^+^).

### Appendix A.3. Structure Verification of ZED1227

The ESI-MS was acquired by injection via LC-MS showing the mass peak of *m/z* 529.37 representing the protonated molecular ion [M + H]^+^, corresponding to the expected molecular mass of 528.6 g/mol.

Structure elucidation by NMR spectroscopy is summarized in Table A1. The chemical structure of ZED1227 with numbering is presented for identification of the chemical shifts.

**Table A1:** ^1^H- and ^13^C-NMR (DMSO-D6), peak assignment of ZED1227.

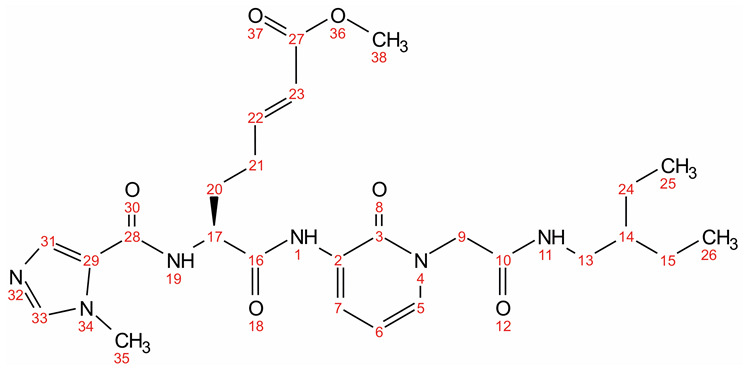

^13^C NMR δ [ppm]	Position	Type	^1^H NMR δ [ppm]
10.75	C32, C33	-CH3	0.82
23.30	C30, C31	-CH2-	1.26
28.30	C20	-CH2-	2.32
29.43	C19	-CH2	1.91, 2.04
33.44	C38	-CH3	3.79
40.43	C14	>CH-	1.26
41.10	C13	-CH2-	3.01
51.18	C26	-CH3	3.62
51.52	C9	-CH2-	4.58
53.18	C16	=CH-	4.58
104.52	C6	=CH-	6.25
121.00	C22	=CH-	5.86
122.34	C7	=CH-	8.21
125.25	C28	=C<	
127.99	C2	=C<	-
133.00	C34	=CH-	7.72
133.50	C5	=CH-	7.33
142.06	C36	=CH-	7.77
148.46	C21	=CH-	6.93
156.69	C3	>C=O	-
160.34	C27	>C=O	-
165.35	C23	>C=O	-
166.77	C10	>C=O	-
170.82	C15	>C=O	-
-	H11	>NH	8.05
-	H18	>NH	8.63
-	H1	>NH	9.29

## References

[1] Sarkar N.K., Clarke D.D., Waelsch H. (1957). An enzymically catalyzed incorporation of amines into proteins. Biochim. Biophys. Acta.

[2] Katt W.P., Antonyak M.A., Cerione R.A. (2018). The diamond anniversary of tissue transglutaminase: A protein of many talents. Drug Discov. Today.

[3] Lorand L., Iismaa S.E. (2019). Transglutaminase diseases: From biochemistry to the bedside. FASEB J..

[4] Szondy Z., Korponay-Szabó I., Király R., Sarang Z., Tsay G.J. (2017). Transglutaminase 2 in human diseases. BioMedicine.

[5] See J., Murray J. (2006). Gluten-Free Diet: The Medical and Nutrition Management of Celiac Disease. Nutr. Clin. Pract..

[6] Lindfors K., Ciacci C., Kurppa K., Lundin K.E.A., Makharia G.K., Mearin M.L., Murray J.A., Verdu E.F., Kaukinen K. (2019). Coeliac disease. Nat. Rev. Dis. Primers.

[7] Kivelä L., Caminero A., Leffler D.A., Pinto-Sanchez M.I., Tye-Din J.A., Lindfors K. (2020). Current and emerging therapies for coeliac disease. Nat. Rev. Gastroenterol. Hepatol..

[8] Kamer J.H.v.d., Weijers H.A., Dicke W.K. (1953). Coeliac Disease: An Investigation into the Injurious Constituents of Wheat in Connection with their Action on Patients with Coeliac Disease. Acta Paediatr..

[9] Bruce S.E., Bjarnason I., Peters T.J. (1985). Human jejunal transglutaminase: Demonstration of activity, enzyme kinetics and substrate specificity with special relation to gliadin and coeliac disease. Clin. Sci..

[10] Dieterich W., Ehnis T., Bauer M., Donner P., Volta U., Riecken E.O., Schuppan D. (1997). Identification of tissue transglutaminase as the autoantigen of celiac disease. Nat. Med..

[11] Molberg Ø., Mcadam S.N., Körner R., Quarsten H., Kristiansen C., Madsen L., Fugger L., Scott H., Norén O., Roepstorff P. (1998). Tissue transglutaminase selectively modifies gliadin peptides that are recognized by gut-derived T cells in celiac disease. Nat. Med..

[12] Sjöström H., Lundin K., Molberg Ø., Körner R., Mcadam S.N., Anthonsen D., Quarsten H., Norén O., Roepstorff P., Thorsby E. (1998). Identification of a Gliadin T-Cell Epitope in Coeliac Disease: General Importance of Gliadin Deamidation for Intestinal T-Cell Recognition. Scand. J. Immunol..

[13] Shan L., Molberg Ø., Parrot I., Hausch F., Filiz F., Gray G.M., Sollid L.M., Khosla C. (2002). Structural Basis for Gluten Intolerance in Celiac Sprue. Science.

[14] Shan L., Qiao S.-W., Arentz-Hansen H., Molberg Ø., Gray G.M., Sollid L.M., Khosla C. (2005). Identification and Analysis of Multivalent Proteolytically Resistant Peptides from Gluten: Implications for Celiac Sprue. J. Proteome Res..

[15] Fleckenstein B., Molberg Ø., Qiao S.-W., Schmid D.G., von der Mülbe F., Elgstøen K., Jung G., Sollid L.M. (2002). Gliadin T cell epitope selection by tissue transglutaminase in Celiac disease - Role of enzyme specificity and pH influence on the transamidation versus deamidation reactions. J. Biol. Chem..

[16] Qiao S.-W., Bergseng E., Molberg Ø., Xia J., Fleckenstein B., Khosla C., Sollid L.M. (2004). Antigen Presentation to Celiac Lesion-Derived T Cells of a 33-Mer Gliadin Peptide Naturally Formed by Gastrointestinal Digestion. J. Immunol..

[17] Bodd M., Tollefsen S., Bergseng E., Lundin K.E., Sollid L.M. (2012). Evidence that HLA-DQ9 confers risk to celiac disease by presence of DQ9-restricted gluten-specific T cells. Hum. Immunol..

[18] Sollid L.M. (2017). The roles of MHC class II genes and post-translational modification in celiac disease. Immunogenetics.

[19] Schuppan D., Dieterich W., Riecken E.O. (1998). Exposing gliadin as a tasty food for lymphocytes. Nat. Med..

[20] Fleckenstein B., Qiao S.-W., Larsen M.R., Jung G., Roepstorff P., Sollid L.M. (2004). Molecular Characterization of Covalent Complexes between Tissue Transglutaminase and Gliadin Peptides. J. Biol. Chem..

[21] Sollid L.M., Molberg Ø., Mcadam S., Lundin K. (1997). Autoantibodies in coeliac disease: Tissue transglutaminase--guilt by association?. Gut.

[22] Iversen R., Du Pré M.F., Di Niro R., Sollid L.M. (2015). Igs as Substrates for Transglutaminase 2: Implications for Autoantibody Production in Celiac Disease. J. Immunol..

[23] Stamnaes J., Iversen R., Du Pré M.F., Chen X., Sollid L.M. (2015). Data from: Enhanced B-cell receptor recognition of the autoantigen transglutaminase 2 by efficient catalytic self-multimerization. PLoS ONE.

[24] Iversen R., Amundsen S.F., Kleppa L., du Pré M.F., Stamnæs J., Sollid L.M. (2020). Evidence That Pathogenic Transglutaminase 2 in Celiac Disease Derives from Enterocytes. Gastroenterology.

[25] Lebreton C., Ménard S., Abed J., Moura I.C., Coppo R., Dugave C., Monteiro R.C., Fricot A., Traore M.G., Griffin M. (2012). Interactions Among Secretory Immunoglobulin A, CD71, and Transglutaminase-2 Affect Permeability of Intestinal Epithelial Cells to Gliadin Peptides. Gastroenterology.

[26] Adamczyk M., Griffiths R., Dewitt S., Knauper V., Aeschlimann D. (2015). P2X7 receptor activation regulates rapid unconventional export of transglutaminase-2. J. Cell Sci..

[27] Stamnaes J., Pinkas D.M., Fleckenstein B., Khosla C., Sollid L.M. (2010). Redox regulation of transglutaminase 2 activity. J. Biol. Chem..

[28] Melkonian A.V., Loppinet E., Martin R., Porteus M., Khosla C. (2021). An Unusual “OR” Gate for Allosteric Regulation of Mammalian Transglutaminase 2 in the Extracellular Matrix. J. Am. Chem. Soc..

[29] Jin X., Stamnæs J., Klöck C., DiRaimondo T.R., Sollid L.M., Khosla C. (2011). Activation of Extracellular Transglutaminase 2 by Thioredoxin. J. Biol. Chem..

[30] Finney S., Seale L., Sawyer R.T., Wallis R.B. (1997). Tridegin, a new peptidic inhibitor of factor XIIIa, from the blood-sucking leech Haementeria ghilianii. Biochem. J..

[31] Reinhardt G. (1981). alpha-Halogenmethyl carbonyl compounds as very potent inhibitors of factor XIIIa in vitro. Ann. N. Y. Acad. Sci..

[32] Shebuski R.J., Sitko G.R., Claremon A.D., Baldwin J.J., Remy D.C., Stern A. (1990). Inhibition of factor XIIIa in a canine model of coronary thrombosis: Effect on reperfusion and acute reocclusion after recombinant tissue-type plasminogen activator. Blood.

[33] Freund K.F., Doshi K.P., Gaul S.L., Claremon D.A., Remy D.C., Baldwin J.J., Pitzenberger S.M., Stern A.M. (1994). Transglutaminase Inhibition by 2-[(2-Oxopropyl)thio]imidazolium Derivatives: Mechanism of Factor XIIIa Inactivation. Biochemistry.

[34] Hausch F., Halttunen T., Mäki M., Khosla C. (2003). Design, Synthesis, and Evaluation of Gluten Peptide Analogs as Selective Inhibitors of Human Tissue Transglutaminase. Chem. Biol..

[35] Sullivan M.P., Nelson J.A., Feldman S., Van Nguyen B. (1988). Pharmacokinetic and phase I study of intravenous DON (6-diazo-5-oxo-L-norleucine) in children. Cancer Chemother. Pharmacol..

[36] Schuppan D., Mäki M., Lundin K.E., Isola J., Friesing-Sosnik T., Taavela J., Popp A., Koskenpato J., Langhorst J., Hovde M. (2021). A Randomized Trial of a Transglutaminase 2 Inhibitor for Celiac Disease. N. Engl. J. Med..

[37] Constantinou-Kokotou V., Magrioti V. (2003). Synthesis and use of *N*,*N*-di-Boc-glutamate gamma-semialdehydes and related aldehydes. Amino Acids.

[38] Kokotos G., Padrón J.M., Martín T., Gibbons W.A., Martín V.S. (1998). A General Approach to the Asymmetric Synthesis of Unsaturated Lipidic α-Amino Acids. The First Synthesis of α-Aminoarachidonic Acid. J. Org. Chem..

[39] Ghosh A.K., Sarkar A., Brindisi M. (2018). The Curtius rearrangement: Mechanistic insight and recent applications in natural product syntheses. Org. Biomol. Chem..

[40] Bharate S.S., Kumar V., Vishwakarma R.A. (2016). Determining Partition Coefficient (Log P), Distribution Coefficient (Log D) and Ionization Constant (pKa) in Early Drug Discovery. Comb. Chem. High Throughput Screen.

[41] Dressman J.B., Krämer J. (2005). Pharmaceutical Dissolution Testing.

[42] Klein S. (2010). The Use of Biorelevant Dissolution Media to Forecast the In Vivo Performance of a Drug. AAPS J..

[43] Ottaviani G., Gosling D.J., Patissier C., Rodde S., Zhou L., Faller B. (2010). What is modulating solubility in simulated intestinal fluids?. Eur. J. Pharm. Sci..

[44] Lorand L., Lockridge O.M., Campbell L.K., Myhrman R., Bruner-Lorand J. (1971). Transamidating enzymes: II. A continuous fluorescent method suited for automating measurements of factor XIII in plasma. Anal. Biochem..

[45] Pasternack R., Büchold C., Jähnig R., Pelzer C., Sommer M., Heil A., Florian P., Nowak G., Gerlach U., Hils M. (2019). Novel inhibitor ZED3197 as potential drug candidate in anticoagulation targeting coagulation FXIIIa (F13a). J. Thromb. Haemost..

[46] Keillor J.W., Hitomi K., Kojima S., Fesus L. (2015). Inhibition of Transglutaminase. Transglutaminases.

[47] Wissner A., Overbeek E., Reich M.F., Floyd M.B., Johnson B.D., Mamuya N., Rosfjord E.C., Discafani C., Davis R., Shi X. (2002). Synthesis and Structure−Activity Relationships of 6,7-Disubstituted 4-Anilinoquinoline-3-carbonitriles. The Design of an Orally Active, Irreversible Inhibitor of the Tyrosine Kinase Activity of the Epidermal Growth Factor Receptor (EGFR) and the Human Epidermal Growth Factor Receptor-2 (HER-2). J. Med. Chem..

[48] Pinkas D.M., Strop P., Brunger A.T., Khosla C. (2007). Transglutaminase 2 undergoes a large conformational change upon activation. PLoS Biol..

[49] Matthews D.A., Dragovich P.S., Webber S.E., Fuhrman S.A., Patick A.K., Zalman L.S., Hendrickson T.F., Love R.A., Prins T.J., Marakovits J.T. (1999). Structure-assisted design of mechanism-based irreversible inhibitors of human rhinovirus 3C protease with potent antiviral activity against multiple rhinovirus serotypes. Proc. Natl. Acad. Sci. USA.

[50] Pedersen L.C., Yee V.C., Bishop P.D., Le Trong I., Teller D.C., Stenkamp R.E. (1994). Transglutaminase factor XIII uses proteinase-like catalytic triad to crosslink macromolecules. Protein Sci..

[51] Santos M.M., Moreira R. (2007). Michael Acceptors as Cysteine Protease Inhibitors. Mini-Rev. Med. Chem..

[52] Zhang L., Lin D., Sun X., Curth U., Drosten C., Sauerhering L., Becker S., Rox K., Hilgenfeld R. (2020). Crystal structure of SARS-CoV-2 main protease provides a basis for design of improved alpha-ketoamide inhibitors. Science.

[53] De Vita E. (2021). 10 years into the resurgence of covalent drugs. Futur. Med. Chem..

[54] De Laurenzi V., Melino G. (2001). Gene Disruption of Tissue Transglutaminase. Mol. Cell. Biol..

[55] Wodtke R., Pietsch M., Löser R. (2020). Solution-phase synthesis of the fluorogenic TGase 2 acyl donor Z-Glu(HMC)-Gly-OH and its use for inhibitor and amine substrate characterisation. Anal. Biochem..

[56] Singh J., Petter R.C., Baillie T.A., Whitty A. (2011). The resurgence of covalent drugs. Nat. Rev. Drug Discov..

